# A comparative analysis of traditional Chinese medicine health management services in Urban versus rural areas of Chongqing: implications for policy and practice

**DOI:** 10.3389/fpubh.2026.1753043

**Published:** 2026-06-26

**Authors:** Chen Liu, Yan Xu, Feng Peng, Feng Wen, Wen Zhao, YiMeng Gao, Hong Tian, Jing Luo

**Affiliations:** 1 Chongqing Banan Traditional Chinese Medicine Hospital, Chongqing, China; 2 Department of Neurology and Psychology, The Fourth Clinical Medical College of Guangzhou University of Chinese Medicine, Shenzhen, China; 3 Department of Neurology, The Second School of Clinical Medicine, Guangzhou University of Chinese Medicine, Guangzhou, China; 4 School of Management, Chongqing University of Technology, Chongqing, China

**Keywords:** comparison between urban and rural areas, grass root public health, improvement strategies, traditional Chinese medicine health management services, urban–rural disparity

## Abstract

**Background:**

Traditional Chinese medicine (TCM) health management plays an increasingly important role in China’s public health system, particularly in community-based and geriatric care. However, significant disparities between urban and rural areas have emerged during the implementation of related policies. TCM services in many cities face multiple challenges at the grassroots, including a shortage of qualified TCM professionals, uneven distribution of healthcare resources, and low utilization rates of available services. Therefore, a comprehensive evaluation of the urban–rural gap in TCM health management services in Chongqing is essential to strengthen local service capacity and inform policy improvement.

**Methods:**

This study, guided by the Donabedian model, developed an evaluation framework for TCM health management services, comprising 19 tertiary indicators across three dimensions: structure, process, and outcome. This model was used to investigate TCM health management services in 10 counties of Chongqing. A total of 566 valid questionnaires were collected, and 30 TCM technicians were recruited to participate in the study.

**Results:**

The overall compliance rate of grassroots TCM health management services in Chongqing was low, with a mean score of 46.22 out of 100. Among the 10 sampled districts/counties, only Fuling District scored above 60. Three indicators (service agreement signing, S1; TCM professionals per 1,000 residents, S4; proportion of TCM professionals among service staff, S6) showed marked inter-district disparities, while the remaining nine indicators exhibited moderate variability. Within the Structure indicators, Fuling District (31.52 points) and Chengkou County (27.86 points) achieved top-tier performance, whereas Youyang County (4.11 points) lagged substantially behind the regional average. Among the P indicators, Xiushan County (20.81 points) and Shizhu County (18.07 points) ranked highest, while Nanchuan District (6.10 points) exhibited notable room for improvement. For the O indicators, Fuling District (22.74 points) and Wanzhou District (21.11 points) attained the strongest scores; in contrast, Xiushan County (6.58 points) recorded the lowest performance.

**Conclusion:**

To address the current challenges and balance the urban–rural gap in of TCM health management services in Chongqing, a multi-pronged strategy is recommended. This strategy should focus on four core areas: (1) earnestly implement the strategy for cultivating talents in TCM; (2) operationalize a “basic service guarantee that follows population mobility” model to dynamically align service availability with residential and occupational migration patterns; (3) strengthen process management and promote differentiated governance strategies between urban and rural areas; (4) enable districts and counties to make precise efforts to strengthen their strengths and address their weaknesses, thereby building a closed-loop management system with clearly defined rights and responsibilities that precisely matches the core needs of the population.

## 1 Introduction

As a core component of the public health system, grassroots public health services play a crucial role in safeguarding people’s health. At the same time, its efficient management serves as a key lever for improving service quality, inherently driving the high-quality development of the grassroots medical system and ultimately having a decisive impact on the implementation of the “Healthy China 2030” strategy (1). Traditional Chinese medicine (TCM) health management services are not only a distinctive feature of China’s medical system but also an important component of the country’s basic public health service system. They were officially incorporated into the national basic public health projects in 2013 and play a significant role in promoting the “Healthy China” initiative (2). This project is a comprehensive health service centered on the concept of “preventing diseases before they occur” in TMC. It aims to help residents enhance their physical fitness, prevent diseases and promote recovery, providing them with all-round and multi-level health services (3).

The management of the capacity of TCM services at the grassroots level is an important guarantee for building a Healthy China and serving the health of the people. In recent years, the introduction of a series of Chinese national policies—from *the Strategic Plan for the Development of TCM (2016–2030)* to *the “14th Five-Year” Action Plan for Enhancing TCM Service Capacity* at the *Grassroots Level*—has underscored the significant role of TCM in healthcare. These documents emphasize the need to continuously improve the quality of TCM services and health management at the primary care level (4, 5). The 15th Five-Year Action Plan explicitly outlines the “*Implementation Plan for Strengthening the Foundation of Medical and Health Services,*” which aiming to optimize the provision of basic services, address the shortcomings in underdeveloped regions and specialties, and promote the equalization of basic medical and health services across urban and rural areas (6). Within this policy framework, achieving equitable access to TCM health management services across urban and rural areas constitutes a strategic imperative.

Currently, there are challenges in grassroots-level TCM services across various cities, including shortages of TCM professionals, uneven regional distribution of resources, insufficient incentives for transferring medical resources to the grassroots level, inadequate promotion of appropriate TCM techniques, and low service utilization rates (7–12). Chongqing has outlined five dimensions of comprehensive coverage for TCM services at the grassroots level in its “Implementation Plan for Enhancing Grassroots TCM Service Capacity (2023–2025)” (13). However, disparities in the implementation and outcomes of this policy persist between urban and rural areas. Grassroots TCM services in Chongqing face heightened challenges attributable to the municipality’s distinctive mountainous topography, which impedes the efficient allocation and integration of medical resources. This geographical constraint not only elevates transportation and time burdens for residents seeking care but also undermines service accessibility and patient adherence.

Therefore, conducting an in-depth investigation into the urban–rural gap in TCM health management services in Chongqing and proposing systematic improvement strategies based on the findings not only enhances the quality and equity of primary-level TCM health management across urban and rural areas in Chongqing, thereby offering a scalable model for TCM health management interventions in remote rural, ethnic minority, mountainous, and reservoir regions characterized by distinct socioeconomic and geographic conditions, but also consolidates Chongqing’s grassroots practices into a “Chinese approach” with global relevance. This study aims to propose strategies for achieving balanced urban–rural health management in Chongqing, providing a reference for developing countries facing similar challenges. It will thus contribute Chinese wisdom and evidence-informed models to the advancement of grassroots health service innovation worldwide.

## 2 Data sources and methods

### 2.1 Data sources

A cross-sectional survey was conducted from July 2024 to July 2025 to assess the current status of TCM health management service capacity in Chongqing, China. A multi-stage, stratified random sampling method was employed to ensure comprehensive regional representation. The municipality was stratified into four major regional clusters based on its official urban planning framework: the Central Urban Area, the Wuling Mountain Area, Qinba Mountain Area and Three Gorges Reservoir Area. A total of 10 districts and counties with high, medium and low Gross Domestic Product (GDP) levels in each region were selected, respectively, (Yubei District, Banan District, Fuling District, Nanchuan District, Wanzhou District, Yunyang County, Chengkou County, Youyang County, Xiushan County, and Shizhu County). The survey targeted three distinct groups: TCM health service administrators, grassroots-level TCM technicians, and residents (with a focus on people aged 65 and above, children aged 0–36 months, and their parents). A simple random sampling method was adopted. Two primary-level medical and health institutions were selected from each sample district and county, and a total of 20 institutions and 30 technical personnel were included (Table 1). Statistical data were extracted from the Chongqing Health Commission Statistical Yearbook.

**Table 1:** The basic information of the survey participants.

The basic information of the interviewed people		Grassroots TMC technicians	
Variable	n(%)	Variable	n(%)
		Age	
Parents of infants aged 0–36 months	94 (16.6)	20–30	13 (43.3)
People over 65 years old and their family members	404 (71.4)	31–40	14(46.6)
Both	68 (12.0)	41–60	3 (10.0)
Gender		Gender	
Male	254 (44.9)	Male	6 (20)
Female	312 (55.1)	Female	24 (80)
Residential address		Residential address	
Banan district	61 (7.0)	Banan district	5 (16.7)
Yubei district	67 (7.7)	Yubei district	2 (6.7)
Nanchuan district	61 (7.0)	Nanchuan district	4 (13.3)
Fuling district	60 (6.9)	Fuling district	3 (10)
Chengkou district	63 (7.3)	Chengkou district	2 (6.7)
Yunyang district	60 (6.9)	Yunyang district	7 (23.3)
Wanzhou district	62 (7.1)	Wanzhou district	2 (6.7)
Shizhu county	60 (6.9)	Shizhu county	2 (6.7)
Youyang county	64 (7.4)	Youyang county	1 (3.3)
Xiushan county	62 (7.1)	Xiushan county	2 (6.7)
Occupation		Education	
Government-affiliated institution staff	10 (1.8)	Master	1 (2.3)
Public institution employee	39 (6.9)	Bachelor degree	22 (72.7)
Employee of a state-owned enterprise	30 (5.3)	Vocational college diploma	7 (25)
private entrepreneur	44 (7.7)	Professional title	
Individual household	118 (20.9)	No rank	3 (10)
Farmer	113 (20.0)	Junior professional title	13 (43.3)
Soldier	3 (0.5)	Intermediate title	10 (33.3)
Others	109 (19.3)	Senior professional title	4 (13.3)

The minimum sample size was calculated using the formula $n=(Z^{2}*P*(1-P))/e^{2}$ . Assuming a 95% confidence level (*Z* = 1.96), a margin of error of 5% (*e* = 0.05), and a population proportion of *p* = 0.5, the estimated minimum sample size was 380. To account for the design effect of multi-stage sampling (set at 1.5) and an anticipated non-response rate of approximately 10%, the target sample size was increased to 640. In practice, over 650 questionnaires were distributed, and 566 valid responses were obtained, yielding an effective response rate of 87.23%. For the questionnaire data, reliability was supported by a Cronbach’s αcoefficient of 0.7467, and validity was indicated by a Kaiser Meyer Olkin (KMO) test coefficient of 0.727. For the semi-structured interviews, reliability was evidenced by a Cronbach’s αcoefficient of 0.9105, and validity by a KMO test coefficient of 0.9576. Data were collected through structured face-to-face interviews conducted by trained investigators in households, ensuring data authenticity and consistency. Additionally, semi-structured telephone interviews were carried out with TCM technicians to deeply explore the obstacles they encountered in service provision, suggestions for improvement, and policy expectations.

### 2.2 Research model

This study employed the internationally recognized Donabedian evaluation framework, known as the Structure-Process-Outcome (SPO) model, a well-established and widely used approach for assessing healthcare system quality (14–19). This evaluation method has also been maturely applied in China (20–22). This model adopts a holistic process-oriented perspective, in which the elements (Structure, S) refer to the static allocation of institutional resources, including human, material, and organizational capacities; the process elements (Process, P) mainly cover the quality or efficiency during the dynamic operation of the institution, focusing on the specific provision and implementation of services; and the outcome elements (Outcome, O) involve the measurement and evaluation of the final outputs and overall effectiveness of the services (23–25).

Applying this model to the evaluation of TCM health management services is highly compatible and necessary. On the one hand, TCM health management services are characterized by long-term engagement, continuity, and a grassroots orientation, necessitating long-term tracking for effective evaluation. On the other hand, as essential public services predominantly delivered through primary healthcare institutions, these services face practical challenges such as high service demand and limited financial investment, making the optimization of service delivery efficiency particularly critical. The model not only aligns with the intrinsic principles of TCM health management, but also enables a systematic evaluation of service outcomes by clarifying the interactions and extent of influence among the three dimensions—structure, process, and outcome—thereby more effectively harnessing the unique advantages of TCM in health management. Moreover, it provides a scientific foundation for optimizing resource allocation and enhancing the quality of TCM health management services in Chongqing.

Based on the Donabedian evaluation framework, this study developed a comprehensive indicator system to assess TCM health management services across three dimensions—Structure (S), Process (P), and Outcome (O)—comprising a total of 19 third-level indicators. This framework is designed to enable a systematic evaluation of the effectiveness of TCM health management services. In this study, structure refers to the foundational conditions necessary for the provision of TCM health management services, encompassing human, financial, and material resources; the quantity and quality of service institutions and facilities, the level of informatization; and policy support mechanisms. Process pertains to the assessment of service delivery quality, including population coverage of TCM health services, management efficiency and continuity, standardization of implementation procedures, regional adaptability, and frequency of technical guidance. Outcomes focus on the extent of service dissemination and the effectiveness of service recipient engagement, evaluated through both objective and subjective dimensions. The subjective dimension emphasizes service recipients’ satisfaction and sense of gain, while the objective dimension assesses the overall performance and operational level of the service system.

The research first followed the grounded theory and conducted semi-structured interviews with the TCM administrative departments providing TCM health management services, grassroots TCM technicians and residents. A qualitative study was conducted on the three links of structure, process and result of TCM health management services. The keywords most frequently mentioned by the visiting and interviewing subjects were selected to construct an index pool for expressing the effectiveness of TCM health management services. Secondly, the expert Delphi method is adopted. Through two rounds of expert scoring, the evaluation index system is determined from the index pool. Finally, based on the index system, questionnaires and interview meetings were designed to collect subjective index data, and research was conducted on departments such as the Chongqing Municipal Health Commission, health centers of various towns and sub-districts, and community health service centers to collect objective index data, which were used for the evaluation of the SPO model.

### 2.3 Evaluation methods

This study employs the entropy method—fuzzy comprehensive evaluation. The entropy method is an objective weighting method based on the theory of information entropy. It achieves weight allocation by calculating the degree of dispersion of the index data. Then, through the method of weighted scoring based on membership degrees, the scores of each index are calculated, and the total scores of each district and county are obtained after summation.

Based on the entropy method, the indicator system is normalized using the principle of maximum optimality to eliminate dimensional differences, enabling the calculation of weights and proportional contributions for each indicator. The resulting proportions for the structure (S), process (P), and outcome (O) dimensions are 35.90, 31.35, 32.76%, respectively. The details of the weight proportion of each level of indicators are shown in Table 2.

**Table 2:** Grade corresponding score calculation table.

Rank	Standardized value interval $\left(X_{i}^{\prime }\right)$	Calculation formula for membership degree $\left(r_{i,k}\right)$
Excellent (*κ* = 1)	[0.8, 1.0]	$r_{i,1}=\{\begin{array}{c} 0\\ \frac{X_{i}^{'}-0.6}{0.2}\\ 1 \end{array}$ $\begin{array}{l} X_{i}^{'}<0.6\\ 0.6\leq X_{i}^{'}<0.8\\ X_{i}^{'}\geq 0.8 \end{array}$
Good (*κ* = 2)	[0.6, 0.8]	$r_{i,2}=\{\begin{array}{c} 0\\ \frac{X_{i}^{'}-0.4}{0.2}\\ 1\\ \frac{0.9-X_{i}^{'}}{0.1} \end{array}$ $\begin{array}{l} X_{i}^{'}<0.4\;\text{or}\;X_{i}^{'}>0.9\\ 0.4\leq X_{i}^{'}<0.6\\ 0.6\leq X_{i}^{'}<0.8\\ 0.8\leq X_{i}^{'}\leq 0.9 \end{array}$
Medium (*κ* = 3)	[0.4, 0.6]	$r_{i,3}=\{\begin{array}{c} 0\\ \frac{X_{i}^{'}-0.3}{0.1}\\ 1\\ \frac{0.7-X_{i}^{'}}{0.1} \end{array}$ $\begin{array}{l} X_{i}^{'}<0.3\;\text{or}\;X_{i}^{'}>0.7\\ 0.3\leq X_{i}^{'}<0.7\\ 0.4\leq X_{i}^{'}<0.6\\ 0.6\leq X_{i}^{'}\leq 0.7 \end{array}$
Qualified (*κ* = 4)	[0.2, 0.4]	$r_{i,4}=\{\begin{array}{c} 0\\ \frac{X_{i}^{'}-0.1}{0.1}\\ 1\\ \frac{0.5-X_{i}^{'}}{0.1} \end{array}$ $\begin{array}{l} X_{i}^{'}<0.1\\ 0.1\leq X_{i}^{'}\leq 0.2\\ X_{i}^{'}>0.2 \end{array}$
Unqualified (*κ* = 5)	[0.0, 0.2]	$r_{i,5}=\{\begin{array}{c} 1\\ \frac{0.2-X_{i}^{'}}{0.1}\\ 0 \end{array}$ $\begin{array}{l} X_{i}^{'}<0.1\;\;\text{or}\;X_{i}^{'}>0.5\\ 0.1\leq X_{i}^{'}<0.2\\ 0.2\leq X_{i}^{'}<0.4\\ 0.4\leq X_{i}^{'}\leq 0.5 \end{array}$

The calculation steps and formulas of the entropy method are follows:

Step1: Data standardization. For positive indicators, the following formula was applied:


$$x_{\mathrm{ij}}=\frac{x_{\mathrm{ij}}-x_{j\min}}{x_{j\max}-x_{j\min}}$$

For negative indicators, the following formula was applied:


$$x_{\mathrm{ij}}^{'}=\frac{x_{j\max}-x_{\mathrm{ij}}}{x_{j\max}-x_{j\min}}$$

Step 2: Calculate the proportion of the i-th sample under the j-th indicator to that indicator:


$$p_{\mathrm{ij}}=\frac{x_{\mathrm{ij}}^{'}}{\sum_{i=1}^{m}x_{i}^{'}}$$

Step 3: Calculate the entropy value of the i-th indicator, where k is a constant, usually taken $\frac{1}{\ln(1)}$ $e_{i}=-k\sum_{i=1}^{m}p_{\mathrm{ij}}\ln p_{\mathrm{ij}}$

Step 4: Calculate the difference coefficient of the i-th indicator: $g_{i}=1-e_{i}$

Step 5: Determine the weight of the i-th indicator:


$$w_{i}=\frac{\kappa g_{i}}{\sum_{i=1}^{n}g_{i}}$$

Step 6: Calculate the comprehensive score:


$$W_{i,k}=W_{i}\times r_{i,k}\times S_{k}$$

Among them, $W_{i,k}$ denotes the weighted membership score of the ii-th indicator with respect to the k -th grade (core intermediate result); $W_{i}$ represents the weight of the ii-th indicator; $r_{i,k}$ is the membership degree of the i-th indicator to the k -th grade, ranging from 0 to 1 and calculated using a trapezoidal fuzzy distribution; $S_{k}$ is the corresponding score assigned to the kk-th grade (as presented in Table 2).

Step 7: Calculate the weighted score of a single indicator:


$$W_{i}^{\mathrm{Su}m}=W_{i}\times (r_{i,1}\times 95+r_{i,2}\times 85+r_{i,3}\times 75+r_{i,4}\times 65+r_{i,5}\times 55)$$

Step 8: Summarize all the indicators to obtain the final 100-point score for each region (Table 3).

**Table 3:** Weight table of each indicator.

First grade indicators	Indicator	Overall indicator combination weight
Structure (S) (0.3589)	Whether to sign the Task Book (S1)	4.417%
	Proportion of grassroots units providing TCM services (%) (S2)	6.093%
	Proportion of village clinics and community health service stations mainly practicing TCM (%) (S3)	2.104%
	Number of TCM practitioners per thousand permanent residents (S4)	9.115%
	The number of full-time guidance personnel for grassroots health guidance classes in districts and counties (S5)	5.880%
	Proportion of TCM personnel in grassroots units providing TCM services (%) (S6)	8.289%
Process (P) (0.31345)	The average increase rate of TCM health management among the older adults over 65 years old (%) (P1)	4.586%
	Average increase rate (%) (P2) of TCM health management for children aged 0–3	7.927%
	The number of times the Grassroots Guidance Section provided guidance on TCM business to grassroots medical and health institutions (P3)	5.387%
	Stability of county-level TCM health service personnel (P4)	3.934%
	Scoring of the guidance process by grassroots TCM health technicians (P5)	5.422%
	The score of the management personnel of the grassroots guidance departments in districts and counties on the effect of TCM health services in towns and sub-districts (P6)	4.089%
Result (O) (0.32757)	The score of the Municipal Health Commission for the work of each district and county (O1)	3.026%
	The forms of TCM health services (O2)	5.255%
	Awareness (O3)	3.534%
	Utilization rate (O4)	5.242%
	Satisfaction (O5)	6.941%
	Accessibility (O6)	4.018%
	Types of services obtained (O7)	4.741%

The coefficient of variation measures the extent of deviation from the equilibrium value. A larger coefficient of variation indicates a greater deviation from the mean, reflecting a higher degree of imbalance. Conversely, a smaller coefficient of variation suggests a smaller deviation from the mean, indicating a more balanced state. For a given indicator, the larger its coefficient of variation across different districts and counties, the lower the degree of equalization in the development of that indicator. The formula is as follows: ${\mathrm{cv}}_{i}=\frac{\sigma }{\mu }\times 100\%$ .

Among these, data with a coefficient of variation (CV) less than 10% exhibit a low degree of dispersion and weak indicator discriminability. A CV between 10 and 20% indicates a moderate degree of dispersion and average discriminability. A CV of 20% or higher reflects a high degree of dispersion and strong discriminability, making such indicators suitable as core evaluation indicators. For detailed results, please refer to Table 4.

**Table 4:** Coefficient of variation table.

Indicator	Weight(%)	Mean	Standard deviation	Variable coefficient(%)
S2	6.093	78.59	6.28	8
S3	2.104	91.34	7.52	8.23
O1	3.026	92.45	7.98	8.63
O3	3.534	91.78	8.25	8.99
S5	5.88	86.42	7.89	9.13
P3	5.387	90.15	8.67	9.62
P6	4.089	89.23	8.74	9.8
O6	4.018	89.87	9.15	10.18
O7	4.741	86.54	8.96	10.35
P1	4.586	88.76	9.21	10.38
O4	5.242	87.65	9.78	11.16
P5	5.422	85.87	9.85	11.47
P4	3.934	87.69	10.12	11.54
O2	5.255	88.96	10.34	11.62
O5	6.941	83.21	14.89	17.9
P2	7.927	84.32	15.78	18.71
S6	8.289	79.85	16.45	20.6
S4	9.115	82.78	18.96	22.9
S1	4.417	85.26	28.45	33.37

## 3 Results

### 3.1 The comprehensive evaluation scores of the effectiveness of TCM health management services in urban and rural areas

The evaluation findings indicated that the effectiveness of grassroots-level TCM health management services across Chongqing’s districts and counties is limited, with an average score of 46.22 points out of 100. Among the 10 sampled districts and counties, only Fuling District scored above 60 points; all others scored below this benchmark (Table 5). The overall compliance rate of TCM health management services at the grassroots-level in Chongqing is low. Moreover, service effectiveness in the core urban metropolitan area significantly exceeds that in non-core urban areas in Chongqing, reflecting persistent urban–rural disparities in both access to and quality of TCM health management services.

**Table 5:** Comprehensive score of each district and county.

Rank	District	Structure (35.89%)	Process (31.345%)	Result (32.757%)	Comprehensive score
1	Fuling district	31.5198	12.8384	22.7402	67.0984
2	Shizhu county	15.9766	18.0719	18.3506	52.3991
3	Yunyang county	22.8525	9.3004	17.7419	49.8948
4	Wanzhou district	14.757	12.5915	21.1069	48.4554
5	Banan district	14.279	16.123	17.9798	48.3817
6	Chengkou county	27.8524	7.749	8.3351	43.9365
7	Nanchuan district	13.3791	6.0972	20.3799	39.8562
8	Yubei district	12.5902	13.7854	12.901	39.2766
9	Xiushan county	10.2707	20.8085	6.5757	37.6549
10	Youyang county	4.1147	16.9987	14.1687	35.2821

### 3.2 The equalization situation of the constituent items of the effect of TCM health management services

From the perspective of evaluation indicators, substantial inter-district and inter-county variability was observed for three indicators: whether a service agreement has been signed (S1), the number of TCM professionals per 1,000 permanent residents (S4), and the proportion of TCM professionals among staff delivering TCM services at grassroots health institutions (S6)—indicating marked imbalances across districts and counties. In contrast, moderate variability was found for the remaining nine indicators (P2, O5, O2, P4, P5, O4, P1, O7, O6), suggesting relatively greater consistency in their implementation across districts and counties. The remaining indicators exhibit limited discriminative power (see Table 4 for details).

As indicated in Table 3, two indicator—snamely, the number of TCM professionals per 1,000 permanent residents (S4) and the proportion of TCM professionals among staff delivering TCM services at grassroots health institutions (S6)—carry the highest weights and demonstrate strong discriminative capacity. These indicators are the primary drivers of inter-district and inter-county variation in the effectiveness of grassroots TCM health management services. Consequently, shortages and uneven regional distribution of qualified TCM professional and technical personnel constitute a critical bottleneck impeding equitable urban–rural development of these services. Moreover, the average annual growth rate (%) of TCM health management services for children aged 0–3 years (P2) and the corresponding service satisfaction rate (O5) are secondary evaluation indicators, exhibiting moderate discriminative power. Both indicators demonstrate statistically significant positive associations with district- and county-level comprehensive performance scores. Notably, the growth rate of children’s TCM health management services and public satisfaction have become key factors constraining the overall effectiveness of urban–rural TCM health management services.

### 3.3 The situation of each link in the S-P-O of TCM health management services

As shown in Table 5, the weight distribution across the three domains—S, P, and O—of TCM health management services is broadly balanced, with the S domain assigned a marginally higher weight. Within the S indicators, Fuling District (31.52 points) and Chengkou County (27.86 points) achieved top-tier performance, whereas Youyang County (4.11 points) lagged substantially behind the regional average. Among the P indicators, Xiushan County (20.81 points) and Shizhu County (18.07 points) ranked highest, while Nanchuan District (6.10 points) exhibited notable room for improvement. For the O indicators, Fuling District (22.74 points) and Wanzhou District (21.11 points) attained the strongest scores; in contrast, Xiushan County (6.58 points) recorded the lowest performance.

## 4 Conclusion analyses

### 4.1 The overall disparity in TCM health services between urban and rural areas at the grassroots level in Chongqing is not obvious

Based on the assessment results, there is no statistically significant urban–rural disparity in the performance of grassroots TCM health services across Chongqing. Notably, Yubei District as the core urban district ranks only 8th in comprehensive TCM health service scores, substantially underperforming remote rural counties such as Shizhu and Yunyang. Rather, it reflects a systemic shortfall: the overall effectiveness of grassroots TCM health services remains low across both urban and rural settings. When applying a 60-point threshold as the minimum benchmark for acceptable performance, only one of the 10 sampled districts and counties meets this standard; the remaining nine fall below it. These findings underscore that the critical constraints facing grassroots TCM health services are not confined to rural areas but stem from pervasive deficiencies.

### 4.2 This creates a paradox of population mobility between urban and rural areas and the coverage of services between urban and rural areas

Population mobility in Chongqing is characterized by a pronounced rural-to-urban migration pattern, with the Main Urban Area serving as the principal demographic magnet. According to the 2020 Seventh National Population Census, the Main Urban Area’s share of Chongqing’s total population reached 65.90%(an increase of 4.73 percentage points from 2010), underscoring a robust and spatially intensified urban concentration trend across all 38 districts and counties. This growth stems primarily from sustained net in-migration from rural areas and geographically peripheral counties, especially into the Central Urban Area, and thus provides empirical validation of China’s ongoing rural-to-urban demographic transition. From 2022 to 2024, Yubei District experienced an average annual growth rate of 5.7% in its permanent resident population aged 0–3 years; however, the coverage rate of standardized TCM health management services for this age group declined by an average of 3.0 percentage points per year.

### 4.3 There are significant differences in TCM health management services between urban and rural areas, and each has its own shortcomings

Urban and rural areas exhibit divergent governance logics in the delivery of TCM services. Cities prioritize outcome-oriented performance, whereas rural areas emphasize resource-based capacity building. The research findings showed that the outcome indicator accounts for 66.67% of the top three highest-scoring indicators in urban areas, thereby constituting a core comparative advantage. This reflects significantly higher levels of public satisfaction, awareness, utilization, and accessibility of TCM services in urban settings compared with rural ones. This disparity is directly attributable to two factors. First, urban residents demonstrate heightened sensitivity to service effectiveness and personal health benefits. Second, the implementation of urban TCM services aligns more strongly with population health needs. In contrast, the outcome indicator represents only 50% of the top three scoring indicators in rural areas. Instead, the structural (S) and personnel (P) indicators dominate, with particularly high scores for S2 (proportion of TCM-dedicated service institutions), S5 (number of full-time TCM guidance personnel at the grassroots level), and S6 (proportion of TCM-specialized personnel among grassroots health workers). Crucially, this rural advantage in structural inputs is not merely an inherited endowment. It is the cumulative result of targeted policy-driven resource allocation, particularly under national initiatives such as the Rural TCM Capacity-Building Project, as well as the functional reconfiguration of the primary care system following sustained out-migration of working-age populations. It therefore serves as the foundational resource base sustaining the grassroots TCM service system.

There remain deficiencies in the input of rural resources. Although rural areas have developed a comparative advantage in resource indicators such as S2, S5, and S6, and are equipped with stable service venues and management personnel, the development of TCM health services continues to be constrained by three core issues: inadequate resource input, underdeveloped institutionalization, and a shortage of professional personnel. Furthermore, the impact of resource-based indicators on service outcomes varies considerably across rural areas. This stands in sharp contrast to the relatively limited influence of such indicators in urban settings. In 2023, the signing rate in rural areas of Chongqing was only 67%, whereas urban areas achieved full (100%) coverage. The lack of institutionalized construction directly resulted in the absence of rigid constraints and standardized guidance for the development of rural TCM services. Consequently, the allocation of policy resources was difficult to translate into tangible service outcomes. Talent serves as the cornerstone of TCM services. In 2023, the number of TCM practitioners per thousand population in rural areas of Chongqing was merely 0.038, whereas that in urban areas reached 0.132. This substantial urban–rural gap in talent availability directly contributes to insufficient professional competency and constrained service quality in rural TCM service delivery.

## 5 Discussion

What Makes Information Useful for Policy Change? Insights from the Measurement for Change (M4C) Framework Applied to Grassroots TCM Services in Chongqing.

The M4C framework posits that evaluation must transcend descriptive problem identification to actively inform and catalyze evidence-informed policy adjustments. A central aim of the M4C approach is to identify the conditions under which information becomes actionable and decision-relevant for stakeholders across multiple governance tiers—from frontline practitioners to senior policymakers. Although our study was not originally conceptualized within the M4C framework, its empirical findings, methodological approach, and policy-oriented recommendations collectively substantiate three foundational determinants of information usefulness: (1) granular disaggregation of data by demographic, geographic, and temporal dimensions; (2) action-oriented interpretive signals that clarify implications for intervention design and implementation; (3) and alignment of feedback loops across administrative, operational, and strategic levels. In the following section, we systematically map our data sources, analytical processes, and key conclusions onto the M4C logic model and discuss their implications for strengthening local public health policy responsiveness and adaptive capacity.

### 5.1 Data-level contribution: granularity and diagnostic utility

Our findings indicated that aggregate composite scores (the overall mean of 46.22/100) provide minimal actionable guidance for policy formulation and implementation. For instance, Xiushan County ranked ninth in the overall score (37.65/100), yet achieved the highest score among process indicators (*p* = 20.81) and the lowest among outcome indicators (*O* = 6.58). A policymaker relying solely on the composite metric would mischaracterize Xiushan as a uniformly underperforming jurisdiction. In contrast, the disaggregated Structure–Process–Outcome (S–P–O) profile (Table 5) reveals a distinct pattern: while process implementation is robust, the county exhibits a critical gap in converting processes into measurable health outcomes—a diagnostic insight that strategically directs interventions toward outcome-aligned supervision mechanisms and service user satisfaction enhancement. Similarly, Fuling District attained the top overall rank (67.10/100), driven primarily by strong structure (*S* = 31.52) and outcome (*O* = 22.74) scores; by contrast, Chengkou County recorded a comparatively high structure score (*S* = 27.85) but a markedly low outcome score (*O* = 8.34). Such nuanced, policy-relevant contrasts are entirely obscured in single-number rankings.

Furthermore, indicators with both high weighting and substantial cross-county variability, specifically S4 (TCM professionals per 1,000 population) and S6 (proportion of TCM staff), generate actionable policy signals. Within the M4C framework, these are leverage indicators: they explain much of the inter-district performance variation and, critically, correspond to a modifiable system lever, namely strategic workforce deployment. The pronounced urban–rural disparity in TCM practitioner density (0.132 vs. 0.038 per 1,000 population) is not merely descriptive; it functions as a change signal that warrants evidence-informed, targeted interventions, including differentiated recruitment incentives, mandatory rural service rotations, and equity-adjusted staffing quotas. Consequently, information products for provincial health authorities should systematically foreground indicators that are both statistically influential and inequitably distributed.

### 5.2 Process-level reflection: the missing feedback loops

The M4C framework emphasizes iterative, stakeholder-engaged feedback between measurement and action, ideally co-produced through sustained collaboration with district-level implementers and decision-makers. While our study employed a cross-sectional design and did not incorporate real-time feedback sessions with district health managers, we acknowledge this as a methodological limitation. Crucially, however, our findings empirically illustrated the functional necessity of such feedback loops for timely policy responsiveness. For example, Yubei District recorded a 5.7% annual growth in its 0–3-year-old population yet concurrently experienced a 3.0 percentage-point decline in TCM service coverage among this demographic. The information remains inert, forfeiting its potential to catalyze timely, evidence-informed corrective actions. To advance M4C-aligned practice, future evaluations must institutionalize longitudinal, multi-level feedback systems: indicator dashboards should be updated biannually (every 3–6 months) and systematically discussed in structured, action-oriented forums. As evidenced across routine health monitoring systems and confirmed by our own process design.

### 5.3 Conclusion and shared learning: three principles of useful information

Our conclusions generate transferable lessons on what makes information useful for resource-constrained, multilayered health systems.

First, action-binding. Useful information must be explicitly to a specific, feasible, and institutionally viable policy lever. Our recommendation to increase recruitment quotas for TCM professionals in low-scoring counties directly links the empirical finding. Namely, the deficit in indicator S4 (TCM professionals per 1,000 population) to an implementable policy instrument. In the absence of such explicit linkage, even highly precise and reliable data risk remaining confined to academic discourse, with limited traction in operational decision-making. This specification strengthens accountability, reduces implementation latency, and embeds responsiveness into the monitoring architecture itself.

Second, level-matching. Information usefulness is inherently tiered, varying systematically according to the decision-making level and functional responsibilities of the intended user. Provincial health planners require comparative equity metrics to inform resource allocation and regional policy calibration. County-level health managers require disaggregated Structure–Process–Outcome profiles (as presented in Table 5) to diagnose specific performance bottlenecks. Frontline primary care facilities, in turn, require granular, real-time process indicators, such as the proportion of children under three receiving standardized TCM-based management protocols to support daily clinical supervision and quality improvement cycles. While our study generated data suitable for all three levels, including definitions, measurement frequency, visualization formats, and dissemination modalities. This participatory design process is essential to prevent misalignment between information outputs and operational decision needs.

Third, dynamic alignment with population mobility. Our identification of a “coverage paradox” rising child population but falling service coverage in Yubei—demonstrates that static denominators yields systematically biased and operationally misleading information. To ensure policy relevance and equity responsiveness, useful information must be grounded in migration-adjusted denominators, updated at least annually, and ideally integrated with real-time civil registration or health insurance enrollment data. Adaptive policy change requires that measurement systems dynamically recalibrate to evolving demographic realities; failure to do so risks misdirecting interventions toward outdated population baselines and perpetuating geographic inequities in service access.

## 6 Recommendations

### 6.1 Steadily advance the strategy for cultivating talents in TCM and enrich the pool of professionals in this field

According to the research results, the shortcomings of TCM health services at the grassroots level exist not only in rural areas but also in inadequate overall service capacity and suboptimal quality. The following three aspects of problems can be prioritized for improvement: First, the number of grassroots-level TCM professional and technical personnel must be expanded. This will be achieved by implementing the “county-level recruitment for grassroots employment” policy for health professionals in districts and counties and by optimizing personnel allocation and retention mechanisms. Furthermore, the regulation mandating that TCM practitioners seeking promotion to senior professional titles complete a minimum period of service in sub-county medical institutions or in designated counterpart-support institutions will be strictly enforced; priority in promotion will be granted to those demonstrating measurable impact in such roles. Second, recruitment quotas for TCM professionals in primary-level health service institutions will be increased, and dedicated TCM talent positions will be established. District- and county-level differentiated talent replenishment plans should be developed based on local profiles, service demand projections, and current TCM human resource gaps. Districts and counties experiencing a per capita shortfall in TCM professionals will be allocated enhanced policy support, including increased recruitment quotas and targeted talent acquisition initiatives. Third, in collaboration with TCM colleges, a special project for cultivating grassroots-level TCM talents has been established. Targeted training programs have been introduced to facilitate graduate employment at the grassroots level. Colleges of TCM have added interdisciplinary courses on TCM health service management and encouraged students majoring in public health management, medicine, and other fields to take minor courses related to TCM. For in-service professionals, specialized training on TCM health service management is organized, industry experts are invited to deliver lectures, and professional competencies are enhanced.

Second, targeted TCM health service initiatives for older adults and children should be prioritized. According to the research data, the prevalence of chronic diseases among older adults is high (with hypertension, diabetes, and other conditions accounting for over 60%), and common pediatric conditions (such as upper respiratory infections and functional gastrointestinal disorders) demonstrate strong demand for TCM therapy. Prioritizing this demographic not only leverages TCM’s preventive strengths and distinctive diagnostic and therapeutic approaches but also generates rapid, visible demonstration effects that catalyze systemic capacity building across grassroots TCM service delivery.

Third, align service delivery with population-specific health demands to enhance public satisfaction with grassroots TCM services. Grassroots institutions should systematically optimize the supply of stratified and differentiated TCM interventions. For children, offer services such as acupoint application and medicated baths; for older adults, provide distinctive TCM rehabilitation and nursing services. Concurrently, regulatory and operational barriers to TCM utilization must be addressed: streamlining procurement and storage protocols for TCM decoction pieces to ensure consistent availability and prescribability at the grassroots level; integrating frequently prescribed TCM decoction pieces and patent medicines into the primary care essential medicines list; establishing a reliable, standardized procurement and distribution mechanism; and piloting regional TCM resource-sharing centers, thereby improving both the equity and timeliness of TCM drug access.

### 6.2 Optimize the allocation of basic public services to align with population mobility

The policy shortcoming that the newly added inflow population has not been effectively absorbed constitutes the core constraint on the equalized development of services. In counties and districts experiencing net population outflow (e.g., Xiushan and Wanzhou), service planning must shift from pursuing absolute population growth to prioritizing foundational stability: “ensuring essential service coverage” and “solidifying the service base.” Service baselines should be scientifically calibrated and dynamically adjusted according to localized demographic profile, including age structure, migration patterns, and resident permanence. Efforts must fully meet the TCM health management needs of current permanent residents; uphold the quality floor of basic public health services; prevent service degradation attributable to population decline; and thereby safeguard the stability and resilience of the service system. Conversely, in areas with net population inflow, the principle of “services following people” must be rigorously implemented. An expansion-target mechanism linking service increments directly to annual net inflow should be established, enabling evidence-based, proportional scaling of TCM health service supply in alignment with demographic growth.

### 6.3 Enhance institutional oversight throughout the governance process and implement spatially differentiated policy approaches for urban and rural areas

In response to the problems such as cities emphasizing results while rural areas rely on resources, and both urban and rural areas being trapped in the shortcoming of process management, as well as the shortage of TCM resources in both urban and rural areas, the following solutions are adopted: First, based on the core advantages and constraints of urban and rural areas, implement a differentiated resource allocation and development strategy of “improving efficiency in cities and strengthening the foundation in rural areas.” For urban areas, further enhance the public’s experience of TCM services. Through online and offline publicity, as well as bringing TCM health science popularization into communities and other means, continuously increase public awareness and utilization. For rural areas, efforts should be made to accelerate the improvement of the service system, promote the signing rate of grassroots TCM health service task books to achieve 100% full coverage, clarify service standards, and transform policy resource inclination into actual service efficiency.

Second, focus on the common shortcomings in process management in urban and rural areas, and take refined supervision, professional guidance and dynamic control as the core to fill the loopholes in the key links of service implementation. Break the fixed supervision model of quarterly or semi-annual periods. Establish a regular supervision system featuring monthly spot checks and dynamic patrols. At the same time, adjust the configuration of supervision subjects, assign dedicated personnel specializing in TCM business to be responsible for supervision, and carry out professional supervision ability training in conjunction to promote the transformation of supervision from “formal inspection” to “professional guidance.”

### 6.4 Enable districts and counties to make precise efforts to strengthen their strengths and address their weaknesses, thereby building a closed-loop management system with clearly defined rights

Counties and districts should adopt a precision-strengthening approach to advance the holistic enhancement of grassroots TCM services. Based on a comprehensive analysis of the performance metrics including strengths (e.g., high-scoring indicators) and weaknesses (e.g., deficit indicators) for each district and county, develop tailored, development strategies, with one distinct policy framework per administrative unit. For example, Yubei District prioritized “increasing TCM workforce proportion and enhancing service satisfaction” by recruiting specialized TCM professionals and streamlining frontline service workflows resulting in measurable gains in both grassroots TCM staffing ratios and public satisfaction indices; Yunyang County has focused on taking the lead in improving the contract-based system for TCM health services. It has strengthened its TCM professional workforce through targeted recruitment and grassroots training, while simultaneously optimizing service delivery to align with the needs of rural residents, thereby enhancing the utilization rate of TCM services.

In summary, this study conducted an in-depth investigation into the implementation status and effectiveness of TCM health management services in Chongqing. It revealed several issues, such as the overall effectiveness of primary-level TCM health services in Chongqing remaining suboptimal. Drawing on the M4C framework, we emphasize that information usefulness requires indicator granularity, actionable signals, and feedback loops to drive policy change. These services face a dual challenge: persistent mismatches between population mobility—particularly urban–rural flow and service coverage. Both urban and rural primary healthcare settings suffer from weak process management systems and systemic shortages of TCM resources, including qualified personnel, standardized diagnostic and therapeutic protocols, and integrated service delivery mechanisms. Targeted interventions are proposed to: (1) earnestly implement the strategy for cultivating talents in TCM; (2) operationalize a “basic service guarantee that follows population mobility” model to dynamically align service availability with residential and occupational migration patterns; (3) strengthen process management and promote differentiated governance strategies between urban and rural areas; (4) enable districts and counties to make precise efforts to strengthen their strengths and address their weaknesses, thereby building a closed-loop management system with clearly defined rights and responsibilities that precisely matches the core needs of the population. These measures not only provide solid data support and feasible pathways for improving local TCM health management services but also offer policy references and actionable insights for many developing countries facing similar challenges.

This study is subject to several limitations. Specifically, the non-probability sampling approach and geographically restricted sample scope constrain the generalizability of the findings, potentially limiting their capacity to capture regional heterogeneity and the nuanced complexities inherent in urban and rural contexts across the city. Future research should address these constraints by employing stratified, multi-stage sampling to broaden geographic and demographic representation, and by incorporating longitudinal designs coupled with multi-dimensional outcome assessments, thereby enabling a more robust examination of the evolving patterns and determinants of TCM service delivery at the grassroots level.

## Data Availability

The raw data supporting the conclusions of this article will be made available by the authors, without undue reservation.
